# Diabetes mellitus is associated with liver metastasis of colorectal cancer through production of biglycan-rich cancer stroma

**DOI:** 10.18632/oncotarget.27674

**Published:** 2020-08-04

**Authors:** Rina Fujiwara-Tani, Takamitsu Sasaki, Kiyomu Fujii, Yi Luo, Takuya Mori, Shingo Kishi, Shiori Mori, Sayako Matsushima-Otsuka, Yukiko Nishiguchi, Kei Goto, Isao Kawahara, Masuo Kondoh, Masayuki Sho, Hiroki Kuniyasu

**Affiliations:** ^1^Department of Molecular Pathology, Nara Medical University, Kashihara, Nara 634-8521, Japan; ^2^Key Laboratory of Neuroregeneration of Jiangsu and Ministry of Education, Co-Innovation Center of Neuroregeneration, Nantong University, Nantong, Jiangsu Province 226001, China; ^3^Drug Innovation Center, Graduate School of Pharmaceutical Sciences, Osaka University, Suita, Osaka 565-0871, Japan; ^4^Department of Surgery, Nara Medical University, Kashihara, Nara 634-8522, Japan

**Keywords:** diabetes, liver metastasis, colorectal cancer, biglycan, mesenchymal stem cell

## Abstract

High morbidity and mortality of cancer, especially colorectal cancer (CRC), in diabetic patients have been reported. In this study, we investigated the relationship between the presence of diabetes mellitus (blood hemoglobin A1C was 6.5% or higher at the time of diagnosis of CRC) and the progression and liver metastasis of CRC. Histopathological findings in the primary lesions, which were preferential to diabetes-complicated CRC (DM-CRC) and the liver metastasis, were also investigated. Of the 473 CRC patients who underwent curative surgical resection, 148 (31%) had diabetes. In DM-CRC cases, the stage was more advanced, with more cases in stage IV or postoperative disease recurrence. Histopathological findings correlated with liver metastasis in DM-CRC, including budding grade, perineural invasion, and myxomatous tumor stroma, and all were highly correlated with the stage. Additionally, myxomatous stroma showed the strongest correlation with liver metastasis in multivariate analysis. Myxomatous stroma in stage III cases correlated with liver recurrence. The myxomatous stroma was abundant in biglycan protein and contained numerous CD90-positive mesenchymal stem cells (MSCs). In human colon cancer cell line HT29, biglycan expression was induced by high sugar concentration, fatty acids, and insulin, and its contact co-culture with MSCs resulted in enhanced stemness and epithelial-mesenchymal transition phenotype. Thus, DM-CRC has higher malignant phenotypes compared to non-DM-CRC, and the involvement of diabetes-induced biglycan may act as a pathogenic factor.

## INTRODUCTION

Diabetes mellitus is a social problem in developed countries due to its frequency and the diversity and severity of its complications. It is estimated that there are about 463 million people with diabetes between the age of 20 and 79 in the world and about 7.39 million in Japan [1]. In recent years, attention has been focused on the relationship between diabetes and cancer, with the latter being one of the various complications observed in patients with diabetes. The incidence of cancer was reported to be higher in diabetic groups than in non-diabetic groups, and the risk of carcinogenesis is increased in early stages of glucose metabolism disorders [2].

People with diabetes have an increased risk of carcinogenesis in most organs [3], including liver, pancreas, colorectum, stomach, breast, lung, oral cavity, and endometrium, and increased risk of associated mortality [2–4]. In particular, pancreatic, liver, and colon cancer are often associated with diabetes [3, 5, 6]. Smoking, diabetes, obesity, and lean meat diet are known as carcinogenic risks in colorectal cancer (CRC) [7]. The meta-analysis of 15 studies found that among 2,593,935 patients with CRC, those with diabetes had a hazard ratio of 1.30 and a mortality rate of 1.26 compared to the non-diabetic patients [8]. Conversely, the incidence of diabetes in CRC is 5–8% [9, 10].

The causes of increased carcinogenic risk in diabetes include increased oxidative stress, high levels of insulin, related growth factors, and its binding factors, insulin receptor substrate-1 and their downstream phosphoinositide 3-kinase (PI3K), AKT, mitogen-activated protein kinase (MAPK) signal, AMP activated kinase (PRKA), mammalian target of rapamycin, sirtuin 1, and autophagy signal activation [2, 4–6, 11–13]. Advanced glycation end-product (AGE), receptor for AGE (RAGE), and high mobility group box-1 (HMGB1) are emphasized as the causes of complications in diabetes [14]. Previously, we reported the promotion of CRC carcinogenesis by AGE-RAGE and HMGB1-RAGE [15, 16]. Furthermore, activation of renin-angiotensin system and aldolase A associated with hyperglycemia promotes CRC progression [17, 18]. Overexpression of biglycan, a class I small leucine-rich repeat proteoglycan (SLRP), is associated with progression, liver metastasis, recurrence and poor prognosis of CRC [19, 20].

Thus, diabetes is considered to be a factor that promotes CRC carcinogenesis and an exacerbation factor. One-fourth of CRC cases with invasion beyond the submucosal layer show liver metastasis during and/or after the operation [21]. One-third of CRC patients died of liver metastasis [22]. Therefore, it is important to elucidate the relationship between diabetes and liver metastasis of CRC. Furthermore, histopathological findings that show the effects of diabetes in CRC or the metastatic ability have not been reported so far. Here, we aimed to clarify the relationship between diabetes and CRC metastasis, especially liver metastasis *via* histopathological examinations.

## RESULTS

### Association of diabetes with liver metastasis in CRC cases

Clinicopathological factors were compared among the 473 CRC cases that had undergone surgical resection (Table 1). There was no difference in local progression (pT) between the two groups; however, lymph node metastasis (pN) and pStage were more advanced in diabetes mellitus-complicated CRC (DM-CRC). In particular, distant metastases were frequently observed in DM-CRC for all liver, peritoneum, and lung metastases.

**Table 1:** Comparison of clinicopathological parameters between non-DM-CRC and DM-CRC

Parameter		Non-diabetic CRC	Diabetic CRC^1^	*P*^4^
*N*		325	148	
Sex	Male	177	75	NS
	Female	148	73	
Age		71 (38–93)	72 (40–95)	NS
Location	Right	119	54	NS
	Left	206	94	
Histology^2^	Differentiated	293	133	NS
	Undifferentiated	32	15	
pT^2^	2	42	25	NS
	3	219	92	
	4	64	31	
pN^2^	0	182	61	0.0030
	1–2	143	87	
Stage^2^	I–II	170	50	0.0015
	III	106	59	
	IV	49	39	
Distant metastasis	Liver	31	30	0.0018
	Peritoneum	5	12	0.0008
	Lung	2	9	0.0007
Recurrence	Negative	314	130	0.001
	Positive	11	18	
Budding grade^3^	1	26	7	0.0002
	2	157	45	
	3	142	96	
Pn^3^	0	236	88	0.0055
	1	89	60	
Ly^3^	0	169	56	0.0054
	1	156	92	
V^3^	0	181	61	0.0040
	1	144	87	
Ex^3^	0	245	98	0.0455
	1	80	50	
Stroma	Usual	245	69	0.0002
	Myxomatous	80	79	

^1^Blood hemoglobin A1C was equal or higher to 6.5% at the time of colorectal cancer (CRC) diagnosis. ^2^Clinicopathological classification is according to UICC-TNM Classification [61]. Differentiated type, pap, tub1, tub2; Undifferentiated type, por, sig, muc; pT2, tumor invades into muscularis propria layer; pT3, tumor invades into subserosa or adventitia; pT4, tumor exposes to the serosal surface or invades other organ; pN0, no lymph node metastasis; pN1–2, metastasis to regional lymph nodes; stage I–II, cases invaded into the submucosal layer or above; stage III, any case with lymph node metastasis; stage IV, any case with or without lymph node metastases but with distant metastases. ^3^Histopathological findings are according to Japanese Classification of Colorectal Carcinoma [62]. Budding grade 1, number of cancer nest of 5 or less cancer cells at the invasive front is 0–4/mm^2^; Budding grade 2, 5–9/mm^2^; Budding grade 3, 10/mm^2^ or more; Pn: 0, no perineural invasion and 1, presence of perineural invasion; Ly: 0, no lymphatic invasion and 1, presence of lymphatic invasion; V: 0, no venous invasion and 1, presence of venous invasion; Ex: 0, no extramural cancer deposits without lymph node structure and 1, presence of extramural cancer deposits without lymph node structure. ^4^ *P* value was calculated by Fisher’s exact test or Chi^2^ test.

### Histological findings associated with liver metastasis in DM-CRC

**Figure 1:**
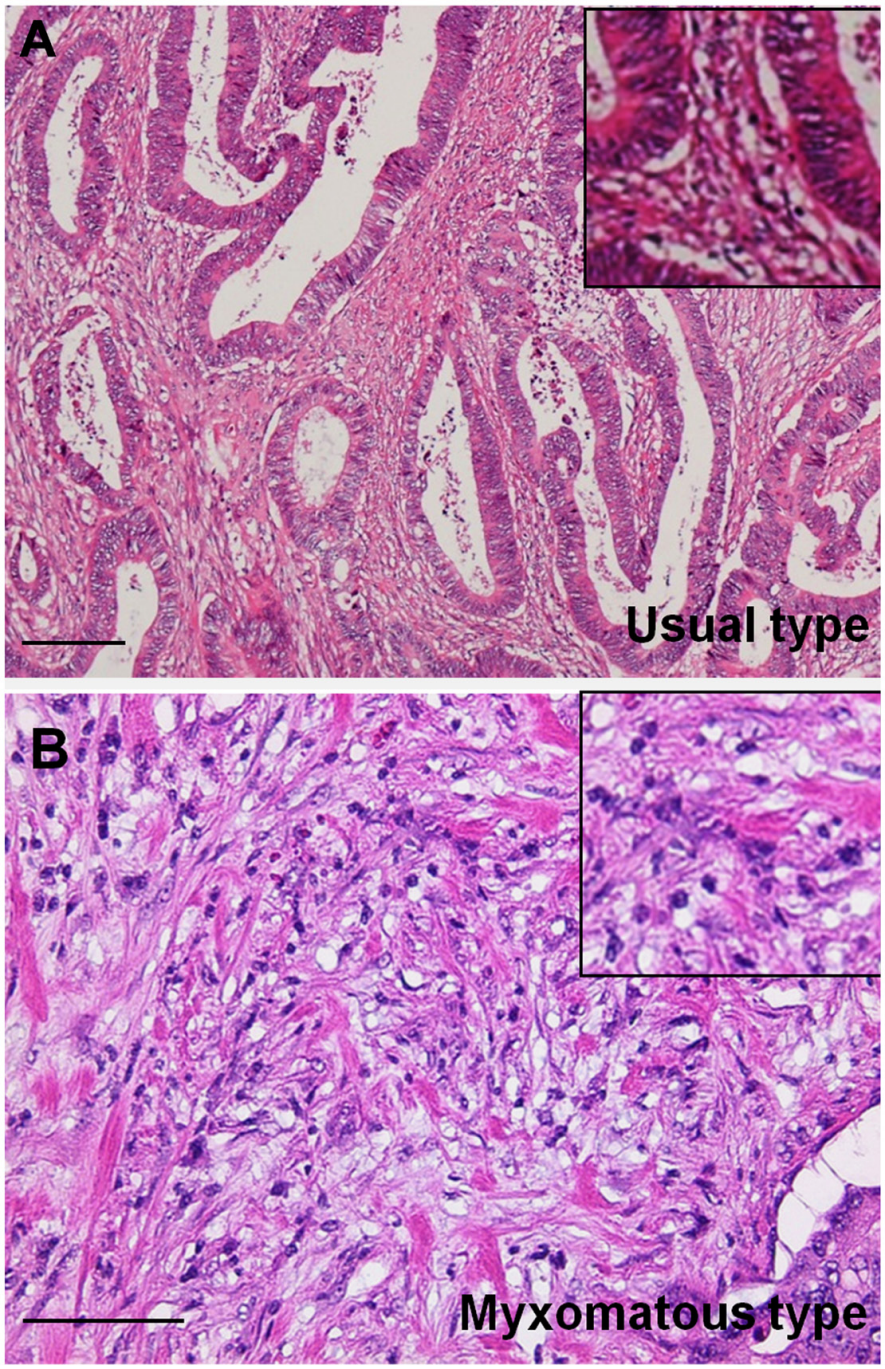
Histopathological findings of myxomatous stroma in colorectal cancer. (**A**) Usual type stroma with abundant collagenous fibers. (**B**) Myxomatous type stroma with weak basophilic stroma resembling cartilaginous mucin with abundant small spindle stromal cells. Inset, high magnification image. Hematoxylin and eosin staining. Scale bar: 100 μm.

Histological findings showed that budding, nerve invasion, vascular invasion, lymph vessel invasion, distant invasion, and myxomatous stroma were observed more frequently in DM-CRC than those in non-DM-CRC. As shown in Figure 1, myxomatous stroma was abundant in stromal cells and poor in collagen fibers, and stromal mucus-like weak basophilic deposits were observed. Furthermore, budding grade, nerve invasion, and the myxomatous stroma were significantly associated with pStage in DM-CRC cases with high significance (Table 2). Furthermore, the correlation between clinicopathological factors and liver metastases was examined by multivariate analysis (Table 3). Myxomatous stroma showed the highest correlation, followed by budding grade.

**Table 2:** Relation of stage with histopathological parameters

		Stage I–II	Stage III	Stage IV	*P*
*N*		220	165	88	
Budding grade	1–2	200	35	0	< 0.0001
	3	20	130	88	
Pn	0	198	113	13	< 0.0001
	1	22	52	75	
Stroma	Usual	208	100	18	< 0.0001
	Myxomatous	12	65	70	

^1^Clinicopathological classification is according to UICC-TNM Classification [61]. Stage I–II, cases invaded into the submucosal layer or above; stage III, any cases with lymph node metastasis; stage IV, any case with or without lymph node metastases but with distant metastases. ^2^Histopathological findings are according to Japanese Classification of Colorectal Carcinoma [62]. Budding grade 1, number of cancer nest of 5 or less cancer cells at the invasive front is 0–4/mm^2^; Budding grade 2, 5–9/mm^2^; Budding grade 3, 10/mm^2^ or more; Pn0, no perineural invasion; Pn1, presence of perineural invasion. ^3^ *P* value was calculated by Chi^2^ test.

**Table 3:** Multiple regression between liver metastasis and pathological parameters

Parameters	Coefficient	95% confidential interval	*P*^5^
Stage^1^	–0.1729	–0.3412–0.004714	0.0438
Subserosal invasion^2^	0.002473	–0.01050–0.01545	0.706
Ly^3^	–0.05134	–0.1400–0.03735	0.253
V^3^	0.04475	–0.01643–0.1059	0.1494
No. of nodal metastasis	–0.01992	–0.04050–0.0006508	0.0573
Budding grade^3^	0.1246	0.02536–0.2239	0.0143
Myxomatous stroma	0.1852	0.04177–0.3287	0.0118
Pn^3^	0.05314	–0.06747–0.1737	0.0384
Ex^3^	0.1869	0.03655–0.3373	0.0152
Mucosal hyperplasia^4^	0.02277	–0.04788–0.09341	0.5236

^1^Pathological stage is according to UICC-TNM Classification [61]. ^2^Distance of subserosal or adventitial invasion from the lower edge of muscularis propria layer (mm). ^3^Histopathological findings are according to Japanese Classification of Colorectal Carcinoma [62]. ^4^Hyperplastic change in the mucosa adjacent to cancer [63, 64]. ^5^ *P* value was calculated by multiple regression analysis using EZR program [60].

### Properties of myxomatous stroma

**Figure 2:**
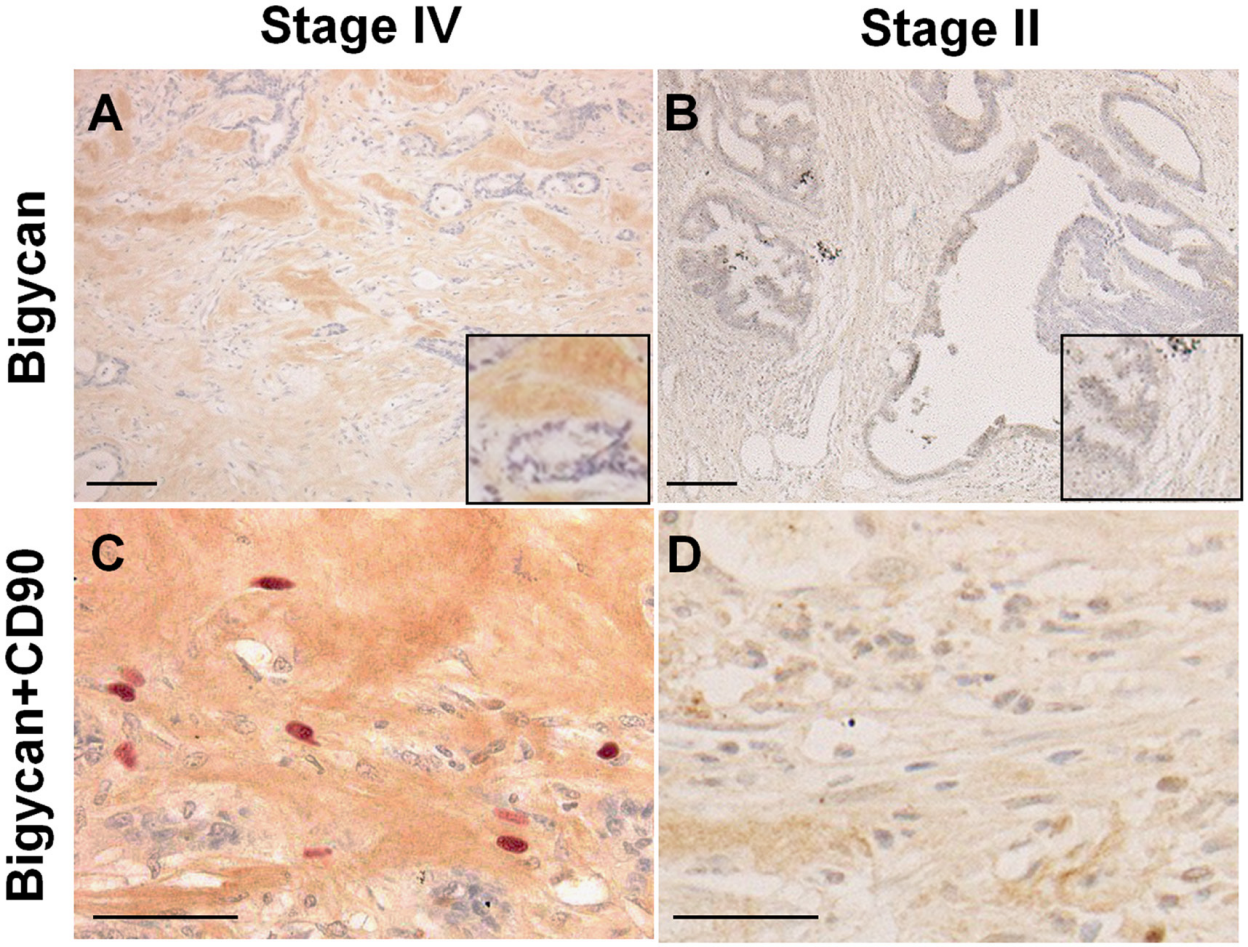
Expression of biglycan and CD90-positive mesenchymal stem cells in cancer stroma. (**A and B**) Immunohistochemistry of biglycan. (**C and D**) Double immunostaining of biglycan (DAB) and CD90 (Fast Red). (A and C) Stage IV colon cancer with liver metastasis. (B and D) Stage II colon cancer without metastasis. Inset, high magnification image. Scale bar: 100 μm.

The properties of myxomatous stroma were examined by immunostaining (Figure 2). The myxomatous stroma showed biglycan expression and the stromal cells comprised of many CD90-positive mesenchymal stem cells (MSCs). Biglycan expression was observed in all cases of myxomatous stroma, except for one case, but only in 11% in case of usual stroma (Table 4). In stage IV CRCs, biglycan expression was observed more frequently in liver metastasis cases (93%) than in non-liver metastasis cases (45%) (Table 5).

**Table 4:** Association of biglycan with myxomatous stroma

Biglycan^1^	Stroma		*P*^2^
	Usual	Myxomatous	
Negative	290	1 (0.3%)	
Positive	36	146 (80%)	< 0.0001

^1^When the biglycan expression levels were higher than those in the mucosa adjacent to cancer, it was judged as overexpression positive. ^2^ *P* value was calculated by Fisher’s exact test.

**Table 5:** Association of biglycan with liver metastasis

Biglycan^1^	Liver metastasis in stage IV		*P*^2^
	Negative	Positive	
Negative	17	2	
Positive	14 (45%)	28 (93%)	< 0.0001

^1^When the biglycan expression levels were higher than those in the mucosa adjacent to cancer, it was judged as overexpression positive. ^2^ *P* value was calculated by Fisher’s exact test.

### Myxomatous stroma predicted postoperative liver metastasis

We examined the relationship between myxomatous stroma and postoperative liver metastasis (liver recurrence) in stage III CRC cases (Table 6). Liver recurrence was found in 7 (7%) of 100 cases with usual stroma, whereas it was found in 19 (29%) of 65 cases with myxomatous stroma. Thus myxomatous stroma might predict liver recurrence in stage III CRCs.

**Table 6:** Association of stroma with postoperative liver metastasis in stage III CRCs

Stroma	Liver metastasis in stage III		*P*^1^
	Negative	Positive	
Usual	93	7 (7%)	
Myxomatous	46	19 (29%)	0.0003

^1^ *P* value was calculated by Fisher’s exact test.

### Mesenchymal stem cells (MSCs) in myxomatous stroma

**Figure 3:**
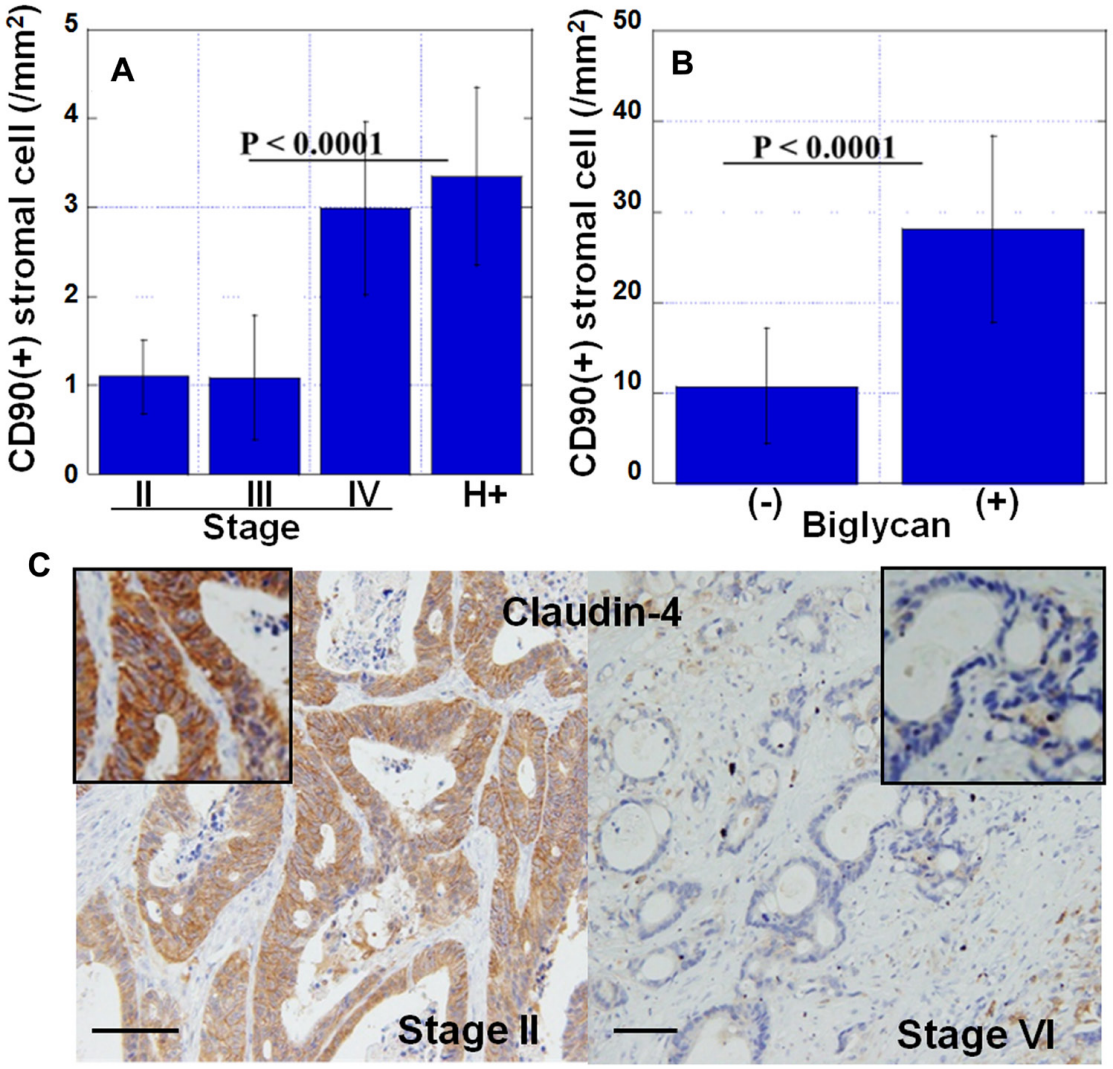
Features of myxomatous stroma. (**A**) Number of CD90-positive mesenchymal stem cells in cancer stroma at the invasive front. H+, stage IV cases with liver metastasis. (**B**) Number of CD90-positive mesenchymal stem cells in biglycan-overexpressed stroma. (+), biglycan overexpression positive; (–), biglycan overexpression negative. (**C**) Expression of claudin-4 in cancer cells in myxomatous stroma (stage IV) or usual stroma (stage II). Inset, high magnification image. Scale bar, 100 μm. Error bar, standard deviation.

Next, we examined the number of CD90-positive MSCs in the stroma in the invasive front (Figure 3). The number of CD90-positive MSCs was significantly higher in pStage IV cases and higher in cases with liver metastases (Figure 3A). Compared with the expression of biglycan, there were significantly more MSCs in biglycan (+) cases than in biglycan (–) cases (Figure 3B). Furthermore, comparison between the expression of biglycan and Claudin-4 in cancer cells showed that the expression of Claudin-4 was significantly decreased in biglycan (+) cases (Figure 3C).

### Relationship between biglycan and epithelial-mesenchymal transition (EMT) in HT29 cells

**Figure 4:**
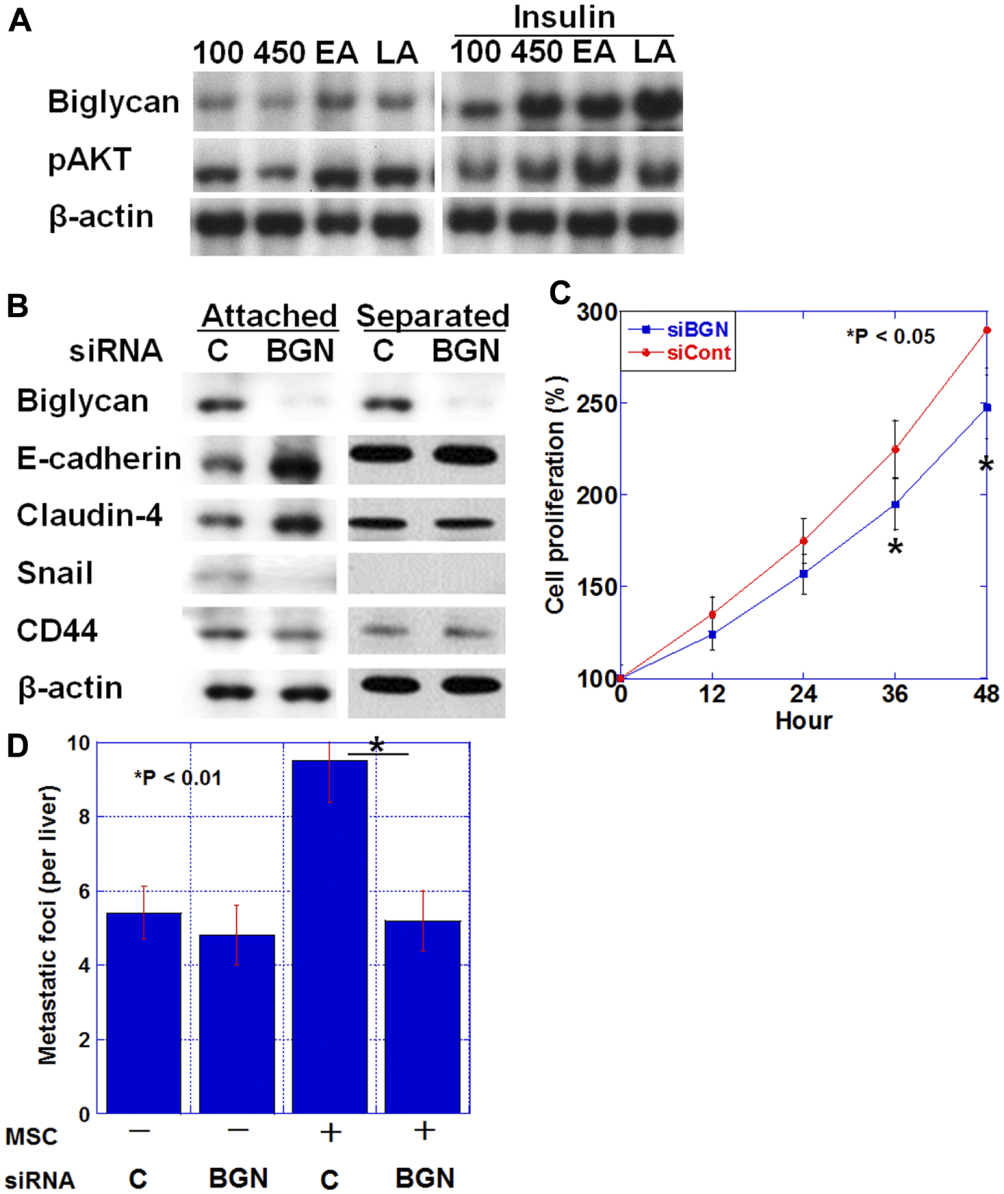
Biglycan expression and epithelial-mesenchymal transition (EMT). (**A**) Biglycan protein expression and phosphorylated AKT level in HT29 cells treated with glucose (100 mg/dL or 450 mg/dL), elaidic acid (70 μM) or linoleic acid (20 μg/ml) and/or insulin (1 μg/mL) for 24 h. (**B**) Effect of biglycan (BGN) siRNA or control siRNA (C) on EMT-associated proteins (E-cadherin, Claudin-4, and Snail) and stemness-associated protein (CD44) in HT29 cells. (**C**) Effect of BGN siRNA (siBGN) or control siRNA (siCont) on cell proliferation of HT29 cells. (**D**) Number of liver metastasis of HT29 human colon cancer cells in nude mice. HT29 cells (1 × 10^6^) pretreated with BGN siRNA or control siRNA (C) were inoculated into the spleen with or without mixed mesenchymal stem cells (MSC, 2 × 10^5^). Error bar, standard deviation.

We examined the effect of diabetes-associated factors on biglycan expression in HT29 human colon cancer cells (Figure 4A). Biglycan protein levels were increased in HT29 cells treated with high level of glucose (450 mg/dL) or the fatty acids, linoleic acid and elaidic acid. Furthermore, simultaneous treatment with insulin synergistically increased the glucose or fatty acid treatment-induced biglycan levels.

Alteration in the expression of EMT-associated proteins was examined when HT29 cells were co-cultured with human MSCs and biglycan was knocked down (Figure 4B). In co-culture conditions where HT29 cells contacted MSCs, biglycan knockdown increased E-cadherin and Claudin-4 expression and decreased Snail and CD44 expression, indicating a reduced EMT phenotype. In contrast, when MSC was placed in an insert chamber and co-cultured with HT29 cells in a non-contact condition, the above alterations in expression were not observed. Biglycan knockdown also decreased cell proliferation in HT29 cells (Figure 4C).

Next, the number of liver metastases was examined when a mixture of HT29 cells and MSCs was inoculated into the spleen of nude mice (Figure 4D). Compared with HT29 alone, the mixed inoculation of HT29 and MSC increased the number of liver metastases by 1.7 fold. In contrast, the mixed inoculation of biglycan knocked down-HT29 cells and MSCs decreased the number of liver metastases to the same level as observed with the inoculation of HT29 alone; this indicated that biglycan knockdown suppressed the effect of the mixed MSCs.

## DISCUSSION

In this study, myxomatous tumor stroma was observed as a histopathological finding that is frequently observed in CRC with diabetes and was shown to have a high correlation with liver metastasis. The myxomatous stroma was rich in extracellular matrix containing biglycan and CD90-positive MSCs.

Biglycan is a component of the cartilagenous matrix [23]. Biglycan is soluble and causes inflammation and autophagy [24, 25]. The stromal mucin-like findings of the myxomatous stroma upon hematoxylin & eosin staining was considered to be due to the nature of the biglycan cartilaginous matrix.

It has been reported that biglycan is overexpressed in various cancers and correlates with progression, metastasis, and angiogenesis of gastric cancer, endometrial cancer, prostate cancer, and bladder cancer [26–31]. In melanoma, biglycan results in stromal stiffness and integrin activation [32]. Biglycan secreted from tumor vascular endothelial cells promotes invasion and metastasis of cancer cells through activation of NFkB and ERK1/2 [33, 34].

Biglycan expression is increased 6-fold in CRC compared with that in normal mucosa [35]. Biglycan expression also increases with progression of CRC and liver metastasis and correlates with recurrence and reduced survival [19, 20]. These findings support the high correlation between biglycan and CRC liver metastasis that we found in the present study.

In our data, knockdown of biglycan reduced stem cell markers and EMT phenotype. Overexpression of biglycan has been reported in spheroids of CD133-positive cancer cells [36], and overexpression in colon cancer stem cells is considered to induce anticancer drug resistance [37]. Biglycan promotes EMT in cancer cells [20, 30], and its expression involves TGFβ/Snail and TNFα/NFkB signals [20, 37]. Thus, high expression of biglycan is associated with high stemness in cancer cells.

In addition, many CD90-positive MSCs were observed in the biglycan-positive myxomatous stroma. Biglycan has been reported to be involved in stemness maintenance of osteoblasts and MSCs by inhibiting their differentiation [38, 39]. It is suggested that undifferentiated MSCs accumulate in the biglycan-positive myxomatous stroma.

MSCs are implicated in many tumor-promoting roles such as angiogenesis, EMT, metastasis, drug resistance, and anti-tumoral immune suppression in cancer [40]. MSCs promote stemness of cancer cells through contact with cancer cells, secretions such as exomes, and fusion with cancer cells [41–43]. Our data suggest that the action of biglycan is mediated by cell contact between cancer cells and MSCs. Such enhancement of EMT and metastatic potential by cell contact between cancer cells and MSCs has been reported in CRC and breast cancer [44, 45]. Upon contact between cancer cells and MSC, their interaction is suspected to occur through soluble bioactive substances and cytoplasmic and organelle interactions [46]. As this effect of MSCs on cancer cells disappears with their differentiation, it is thought that the maintenance of MSC stemness by biglycan is important for acquiring the metastatic potential of cancer cells.

In this study, biglycan overexpression was frequently observed in DM-CRC. Our data also showed that insulin induced biglycan production, with synergistic effects observed with simultaneous treatment with high concentration of glucose and pro-tumorous fatty acids, linoleic acid and elaidic acid [15, 16, 47–51]. Such high levels of insulin, glucose, and fatty acids are associated with diabetic condition. There are many reports on the overexpression of biglycan and the promotion of diabetic complications in diabetes patients. In diabetes, biglycan expression in aortic stromal cells is increased and promotes its destruction [52]. Biglycan expression is also increased in adipocytes in diabetes and is related to insulin resistance and fat inflammation [53]. In renal glomeruli, biglycan expression increases in diabetes and correlates with the development of diabetic nephropathy. TGFβ and PDGF have been reported to promote biglycan expression through AKT activation [54]. It is considered that biglycan expression is promoted by AKT activation even in diabetes showing hyperinsulinemia, which activates AKT [55, 56]. On the other hand, biglycan expression is induced in tumor vascular endothelial cells by promoter demethylation [33].

In this study, diabetes was defined as a blood hemoglobin A1C of 6.5% or higher at the time of CRC diagnosis. It was not possible to examine the timing of diabetes diagnosis or the treatment. It is thought that the role of diabetes on the malignant phenotype of CRC could be examined in more detail by examining the duration of diabetes, the control status of blood sugar, and the treatment content. However, blood hemoglobin A1C 6.5% at the time of CRC diagnosis was shown to be a higher blood glucose level than that at least in healthy individuals, which is considered as a potential indicator of diabetic metabolic disorder. The biglycan overexpression, which is thought to be associated with hyperinsulinemia, might also be considered as evidence of diabetes-related metabolic disorders. In future, it would be desirable to investigate the relationship with cancer, taking into account more detailed studies on diabetes, which will increase the importance of our data.

In conclusion, our study suggests that diabetes promotes liver metastasis of CRC *via* biglycan, which induces cancer stemness and EMT from interaction with MSCs. This novel mechanism is believed to promote cancer malignancy in various cancers complicated with diabetes. The results of our study indicate the importance of diabetes management in malignant tumors in diabetes patients. And it is emphasized the significance of biglycan as a hopeful therapeutic target in malignant tumors with diabetes.

## MATERIALS AND METHODS

### Surgical specimens

We reviewed the pathological diagnosis and clinical data of 473 patients with surgically resected CRC, diagnosed at the Department of Molecular Pathology, Nara Medical University from 2006 to 2015. We used all cases above for analysis in this study without any selection. As written informed consent was not obtained, any identifying information was removed from the samples prior to analysis, in order to ensure strict privacy protection (unlinkable anonymization). All procedures were performed in accordance with the Ethical Guidelines for Human Genome/Gene Research enacted by the Japanese Government and were approved by the Ethics Committee of Nara Medical University (Approval Number 937).

### Cell lines and reagents

HT29 human colon cancer cell line was purchased from Dainihon Pharmaceutical Co. (Tokyo, Japan). Human bone marrow-derived mesenchymal stem cell line (hMSC-BM) was purchased from Takara Bio (Kusatsu, Japan). Cells were cultured in Dulbecco’s modified Eagle’s medium supplemented with 10% fetal bovine serum at 37°C in 5% CO_2_.

For the attached co-culture of HT29 and hMSC-BM cells, HT29 cells (1 × 10^4^) were mixed with hMSC-BM cells (2 × 10^3^) and cultured for 24 h in a 24-well dish. For the separated co-culture of the two cell lines, HT29 (1 × 10^4^) cells were seeded on the bottom of a 24-well dish and hMSC-BM cells (2 × 10^3^) were seeded in an insert with 3 μm-pore (Thermo Fisher Scientific, Waltham, MA, USA) for 24 h. HT29 cells were pretreated with the siRNA for biglycan or control. HT29 cells were separated from the MSCs with EasySep Human EpCAM Positive Selection Kit II (Veritas Corp., Tokyo, Japan) and were subjected to further examination.

Linoleic acid (20 μg/mL, Sigma), elaidic acid (70 μM, Wako Pure Chemicals, Osaka, Japan), and insulin (1 μg/mL, Wako) were used for cell treatments.

### Animals

BALB/c nude mice (4-weeks-old, male) were purchased from SLC Japan (Shizuoka, Japan). The mice were maintained according to the institutional guidelines approved by the Committee for Animal Experimentation of Nara Medical University, in accordance with the current regulations and standards of the Ministry of Health, Labor, and Welfare (Approval number 12262).

To establish a liver metastasis model, HT29 cells (1 × 10^6^) were inoculated into the spleen of nude mice. Then, with five mice in each group, pretreatment was carried out with siRNA for human biglycan for 24 h and/or co-inoculation with hMSC-BM cells (2 × 10^5^ cells). The livers were sectioned into 2-mm-thick slices, and metastatic foci were counted using a stereomicroscope (Nikon, Tokyo, Japan).

### Immunohistochemistry

Consecutive 4-mm sections were immunohistochemically stained using anti-biglycan mouse monoclonal antibody (0.2 e mon, clone 3E2, Santa Cruz Biotechnology, Santa Cruz, CA, USA) and anti-CD90 rabbit monoclonal antibody (0.2 d ant, clone EPR2959, Abcam plc., Cambridge, UK) or anti-CLDN4 antibody (0.2 μg/mL, clone 4D3), which was established in our laboratory [57], and a previously described immunoperoxidase technique [58]. Secondary antibodies for peroxidase-conjugated mouse IgG and alkaline phosphatase-conjugated rabbit IgG (Medical and Biological Laboratories, Nagoya, Japan) were used at a concentration of 0.2 μg/mL. Tissue sections were color-developed with diamine benzidine hydrochloride (DAKO, Glastrup, Denmark) for biglycan and with fast red (CosmoBio, Tokyo, Japan) for CD90. Slides were counterstained with Meyer’s hematoxylin (Sigma). Overexpression of biglycan was determined when the expression was stronger than that of biglycan in normal colon mucosa. For evaluation of CD90 immunostaining, number of positive cells was counted in 500 cells. For negative control, non-immunized rat IgG (Santa Cruz) was used as a primary antibody. Positive straining for biglycan was defined as stronger staining than that in normal colonic epithelium. We used placental tissue as a positive control.

### Immunoblot analysis

Whole-cell lysates were prepared as previously described [59]. Lysates (20 μg) were subjected to immunoblot analysis using SDS-PAGE (12.5%), followed by electrotransfer onto nitrocellulose filters. The filters were incubated with primary antibodies, followed by peroxidase-conjugated IgG antibodies (Medical and Biological Laboratories). Anti-tubulin antibody was used to assess the protein levels loaded per lane (Oncogene Research Products, Cambridge, MA, USA). The immune complex was visualized using an Enhanced Chemiluminescence Western-blot detection system (Amersham, Aylesbury, UK). Antibodies for biglycan (Santa Cruz), phosphorylated AKT (phosphoSer473, Proteintech Group Inc. Rosemont, IL, USA), E-cadherin (DAKO), CLDN4 (clone 4D3) [57], Snail (Biorbyt, St Louis, MO, USA), and CD44 (Abcam) were used as primary antibodies. β-actin, detected by antibody (Abcam), was used as the loading control.

### Short interfering RNA (siRNA) assay

FlexiTube siRNAs targeting human biglycan gene (*BGN*) were purchased from Santa Cruz Biotechnology. AllStars Negative Control siRNA (Qiagen) was used as a control. Cells were transfected with 50 nM siRNA using Lipofectamine 2000 (Invitrogen, Carlsbad, CA, USA), according to the manufacturer’s instructions.

### Statistical analysis

Statistical significance was calculated using a two-tailed Fisher’s exact test, an ordinary ANOVA, and InStat software (GraphPad, Los Angeles, CA, USA). Multiple regression analysis was performed using EZR program [60]. A two-sided *P* value of < 0.05 was considered to indicate statistical significance.
